# Rhomboid blepharoplasty and cryotherapy for the treatment of a squamous cell carcinoma on the lower eyelid in a horse

**DOI:** 10.1002/ccr3.1907

**Published:** 2018-11-11

**Authors:** Emily C. Jeanes, Sarah Koll‐Hampp, Charlotte Dawson, Bettina Dunkel, Roser Tetas Pont

**Affiliations:** ^1^ Centre for Small Animal Studies Animal Health Trust Newmarket, Suffolk UK; ^2^ Queen Mother Hospital for Animals Royal Veterinary College North Mymms, Hertfordshire UK; ^3^ Equine Hospital Royal Veterinary College North Mymms, Hertfordshire UK

**Keywords:** blepharoplasty, equine, rhomboid flap, squamous cell carcinoma

## Abstract

A rhomboid blepharoplasty can be used to achieve functional and cosmetic eyelid reconstruction at the medial canthus in the horse. Combination of a rhomboid blepharoplasty with cryotherapy is a treatment option for eyelid ocular squamous cell carcinomas.

## 1 INTRODUCTION

A 9‐year‐old gelding presented with a medial lower eyelid squamous cell carcinoma. The tumor was resected “en‐bloc” and the margins treated with cryotherapy under general anesthetic. Reconstruction of the lower eyelid was achieved using a rhomboid blepharoplasty. In 12 months’ follow‐up period, no recurrence of the tumor was noted.

Squamous cell carcinoma (SCC) is the most common tumor of the equine orbit and adnexa and the second most common tumor in the horse overall.^1^ The prevalence of ocular SCC in horses is positively correlated with increased exposure to ultraviolet light.^2^ Lack of skin pigmentation also increases the risk of becoming affected.^2^ Draught horses, the Haflinger breed, and Appaloosa horses have been shown to be predisposed to SCC development.^2^, ^3^, ^4^ A mutation in the *damage‐specific DNA‐binding protein 2* (*DDB2*) has been identified which increases the risk of developing SCC of the limbus and nictitans membrane in horses, specifically in the Haflinger and Belgium breeds.^5^, ^6^ A gender predilection has also been proposed with castrated males being overrepresented,^2^, ^7^, ^8^ although this is not supported by other studies. This epithelial neoplasm in horses has been described originating from the cornea, limbus, conjunctiva, nictitans membrane, and eyelid.^3^, ^7^, ^8^, ^9^, ^10^ Assessing the numbers from all these studies together shows that the most common site appears to be the nictitans membrane (39%) and the second most common site the eyelids (29%).^3^, ^7^, ^8^, ^9^, ^10^

Ocular SCCs are typically highly locally invasive but the reported metastatic rate is low.^8^ Eyelid SCC has been shown to carry a poorer prognosis compared to other locations.^10^ The disease is usually unilateral, although bilateral squamous cell carcinomas have been described.^8^, ^11^ The mainstay of treatment of eyelid SCC is surgical excision.^12^ Recurrence after surgical excision is likely, particularly if clear surgical margins cannot be achieved.^13^ The recurrence rate reported varies and adjuvant therapy, such as cryotherapy, radiofrequency hyperthermia, radiotherapy, topical and intralesional chemotherapy with cisplatin or 5‐fluorouracil, bacille Calmette‐Guerin (BCG) cell wall extract, and carbon dioxide laser ablation is often considered to avoid further recurrences.^1^, ^12^

When eyelid surgery is considered, it is important to preserve enough of the healthy tissue to maintain normal eyelid function.^1^ Blepharoplasties in horses are rarely reported in the literature due to the challenges of sliding or moving the poorly mobile tissue around the horse eye^1^, ^14^; subsequently, enucleation or exenteration may be required at the expense of a visual eye.^15^ The rhomboid blepharoplasty has been described for reconstruction of the medial canthus in the human^16^ and was described in one horse for reconstruction of the lateral canthus.^17^ This case report describes the treatment of a lower eyelid SCC in a horse with a rhomboid blepharoplasty procedure and cryotherapy with no recurrence in a 12‐month follow‐up.

## 2 CASE REPORT

Ethical approval for this study was granted by the Royal Veterinary College's Social Sciences Research Ethical Review Board (URN SR2017‐1088).

A 9‐year‐old Irish Draught gray gelding was referred to the Ophthalmology Department at the Royal Veterinary College (RVC) for the evaluation and treatment of an ocular SCC on the lower right eyelid in April 2017. The tumor had been diagnosed previously by histopathological examination of a biopsy from a 6x4mm mass taken by the referring veterinary surgeon.

### 2.1 Initial ophthalmic examination

The horse presented to the RVC 18 days after the initial biopsy was taken. The patient was bright, alert, and responsive. He was in good body condition and appeared to be in good overall health. Physical examination was unremarkable except for mild enlargement of the left submandibular lymph node. The right submandibular lymph node was unremarkable on palpation.

**Figure 1:**
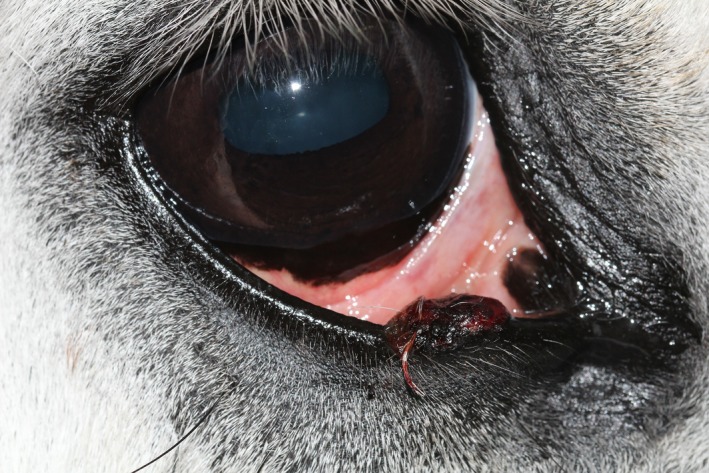
Right eye at presentation with the eyelids in a physiologic position. Please note the lower eyelid mass affecting the medial part of the eyelid

**Figure 2:**
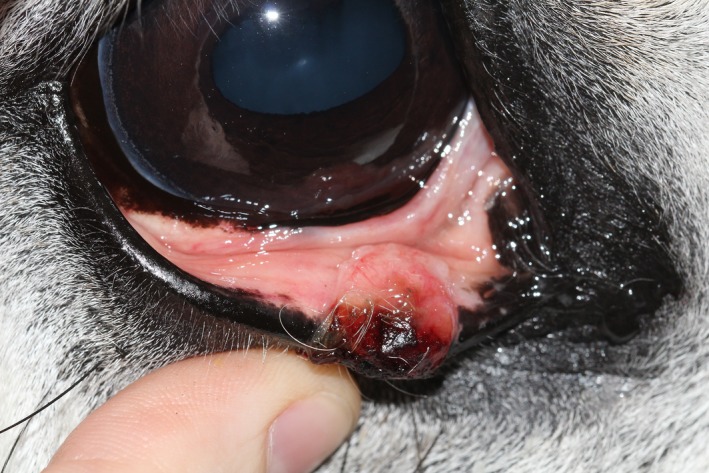
Right eye at presentation with the lower eyelid everted to show the extent of the mass at the conjunctival side of the eyelid

The patient was visual, with intact positive menace responses, dazzle and pupillary light reflexes in both eyes. A 10 × 12 mm large, pigmented, raised, firm mass with an ulcerated hemorrhagic surface was present on the lower eyelid approximately 10 mm from the medial canthus (Figure 1). This mass was bulging from the medial lower eyelid margin and extending 5 mm into the conjunctival side of the eyelid (Figure 2). The surrounding conjunctiva and sclera were mildly hyperemic, and unilateral epiphora was noted. The surfaces of the left and right third eyelid were slightly irregular with a granular appearance. No abnormalities were detected on a complete examination of both globes using slit lamp biomicroscopy (Sl‐15; Kowa Ltd., Tokyo, Japan), and direct (Keeler Professional; Keeler Ltd., Windsor, UK) and indirect ophthalmoscopy (Keeler Vantage Plus; Keeler Ltd.). Intraocular pressure was estimated via rebound tonometry (TonoVet^®^; Icare Finland Oy, Helsinki, Finland) and was within normal limits.

### 2.2 Clinical management

Surgical resection of the SCC and reconstruction of the eyelid under general anesthesia was planned for the following day at the RVC.

Surgery (“en‐bloc” excision, cryotherapy of the eyelid margins, and rhomboid blepharoplasty).

**Figure 3:**
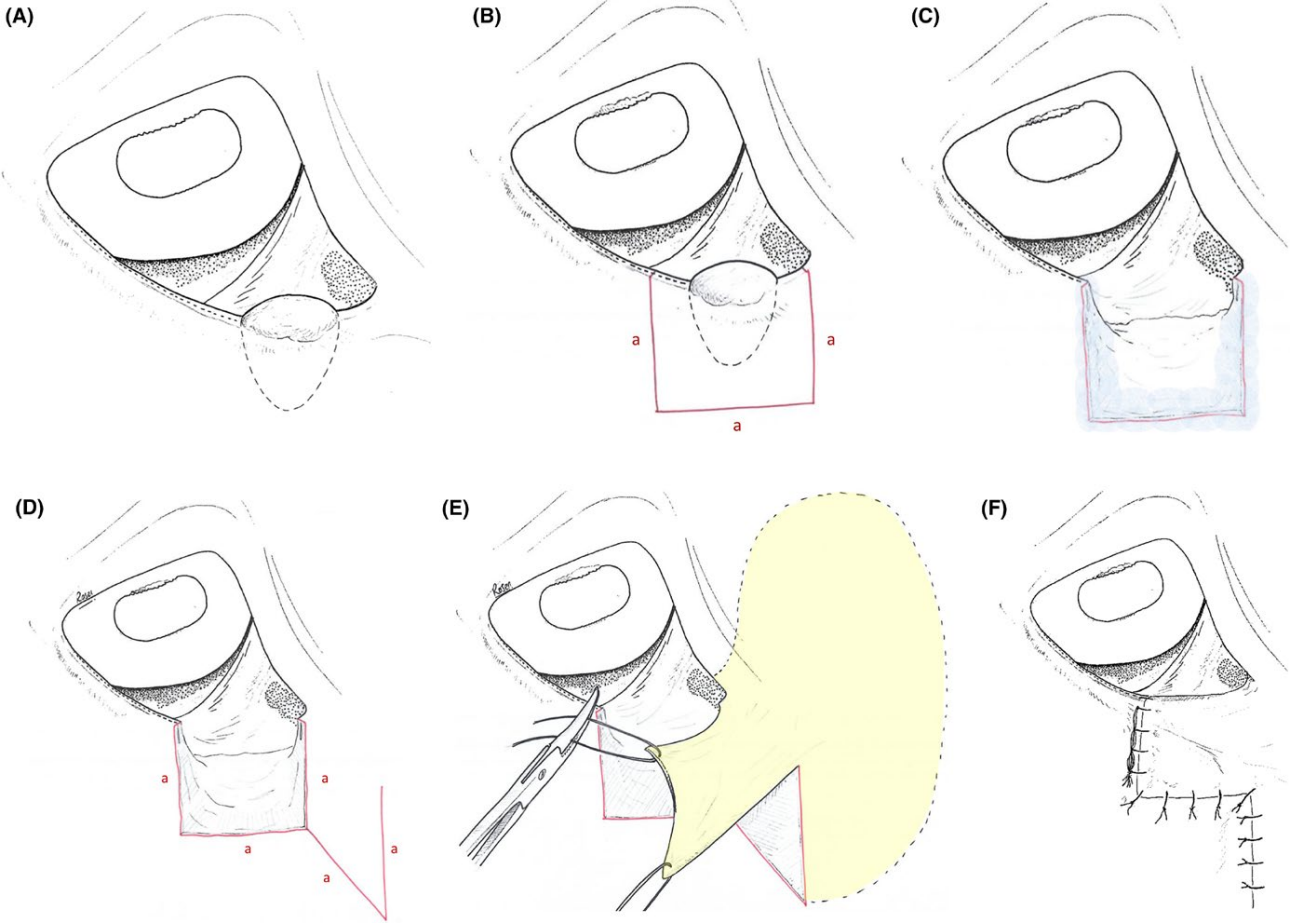
Diagram showing the surgical procedure. A, The eyelid mass before surgery. Figure B, 5 mm margins for excision outlined. C, Full‐thickness excision of the eyelid mass. D, Rhomboid flap outlined. E, Undermining of the periocular skin (shaded yellow) to allow adequate movement of the flap. F, The rhomboid flap in position. It is then sutured in place

The patient was sedated using detomidine (Domidine; Dechra Veterinary Products, Shrewsbury, UK) 0.03 mg/kg and morphine (Martindale Pharma, Buckinghamshire, Shrewsbury, UK) 0.1 mg/kg IV. General anesthesia was induced with ketamine (Ketamidor^®^; Chanelle, Hungerford, UK) 2.2 mg/kg and midazolam (Hypnovel; Roche products Ltd, Welwyn Garden City, UK) 0.05 mg/kg IV followed by endotracheal intubation. General anesthesia was maintained using inhalational isoflurane (Isoflo; ABBOTT, Lake Bluff, IL, USA). The patient was positioned in left lateral recumbency. The lower eyelid of the right eye and medial canthus were clipped. The clipped margins extended 10 cm ventral to the lower eyelid and 15 cm medial to the medial canthus. Sterile preparation of the surgical field was performed with 1:10 Povidone Iodine: sterile 0.9% NaCl. The ocular surface was cleaned with 1:50 Povidone Iodine: sterile 0.9% NaCl. Full‐thickness skin incisions were made through the eyelid 5 mm medial, lateral and ventral to the mass with a number 15 blade (Swann Morton Ltd., Sheffield, UK) and Stevens tenotomy scissors, to achieve en‐bloc excision of the tumor (Figure 3B). These incisions were all of the same length so that a square of tissue was removed. Approximately, a third of the lower eyelid length was excised en‐block. The nasolacrimal apparatus was sacrificed and no attempt was made to preserve it. Hemostasis was achieved using handheld cautery (EYE CAUTERY; John Weiss & Son Ltd, Milton Keynes, UK). A handheld cryotherapy spray (CRYOSPRAY 59; Cuxon Gerrard & Co. Ltd., Oldbury, UK) was used to apply liquid nitrogen to the margins (Figure 3C). Two freeze‐thaw cycles were used of 10 seconds freezing then 60 seconds thawing. An incision was made extending ventromedially from the ventromedial aspect of the excision site. This incision was the same length as the previous incisions. A proximal vertical incision was then made from the ventromedial edge of the last incision to create a rhomboid‐shaped skin graft (Figure 3D). Blunt dissection was used to undermine the rhomboid graft and the skin medial to the graft to mobilize the tissue. The rhomboid graft was swung dorsally and laterally to fill the eyelid defect (Figure 3E). Extensive blunt dissection to undermine the subcutaneous tissue medially, ventrally and dorsally around the skin flap was required to achieve this degree of mobility. Subcutaneous sutures were placed to release tension using 4‐0 polydiaxanone (PDS^®^; Ethicon, Diegem, Belgium) in a simple interrupted pattern. A figure of eight suture with 6‐0 Polyglactin 910 (Vicryl^®^; Ethicon) was used to oppose the eyelid and the graft at the eyelid margin. Intradermal sutures (6‐0 Vicryl^®^; Ethicon) were used to close the skin, leaving a “Z” shaped pattern (Figure 3F). A temporary tarsorrhaphy was placed at the medial canthus using two simple interrupted sutures to protect the graft from movement. Conjunctival biopsies were taken from the upper eyelid, lower eyelid and nictitating membrane of both eyes. A fine needle aspirate of the left submandibular lymph node was taken and submitted for cytology. The excised tissue, along with the conjunctival biopsies and submandibular aspirate was submitted for histopathologic examination. An eye mask was placed to protect the surgery site from trauma during the postoperative period. Recovery from general anesthesia was uneventful.

### 2.3 Laboratory results

**Figure 4:**
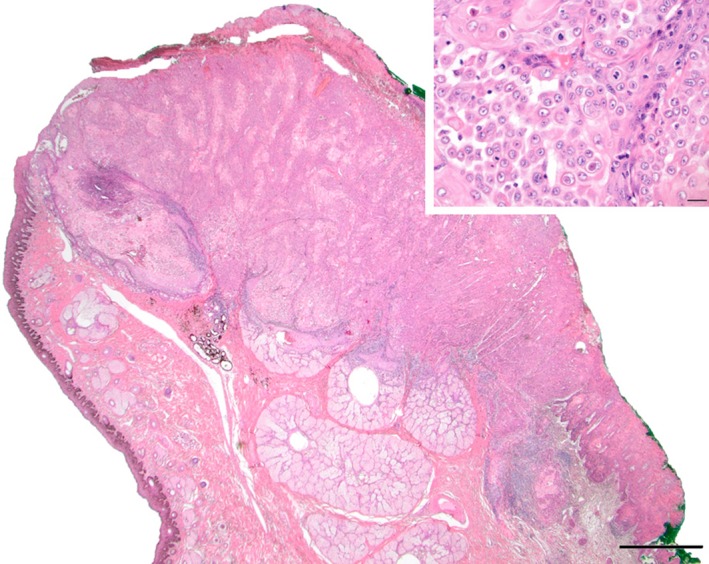
Hematoxylin and eosin showing a poorly differentiated squamous cell carcinoma. The tumor is effacing and ulcerating the conjunctiva and deeply infiltrates the subjacent fibroconnective tissue. There is destruction of Meibomian glands and secondary lymphoplasmacytic inflammation. Scale bar 1 mm. *Inset*: Neoplastic cells are often dyskeratotic and acantholytic. Note bizarre mitotic figures and cell cannibalism

Histopathology of the eyelid mass revealed an infiltrative densely cellular neoplasm of epithelial cells, consistent with a SCC (Figure 4). Tumor‐free margins were identified; however, neoplastic cell emboli were identified in lymphatic vessels within the subconjunctival connective tissue deep to the mass. This suggested the possibility of future extension of the neoplasm to regional lymph nodes. Histopathology of the conjunctival biopsies revealed multifocal chronic lymphofollicular conjunctivitis, consistent with chronic inflammation. Cytology of the submandibular lymph node revealed a mildly reactive mixed lymphoid population. No evidence of metastatic SCC was identified.

### 2.4 Follow‐up

**Figure 5:**
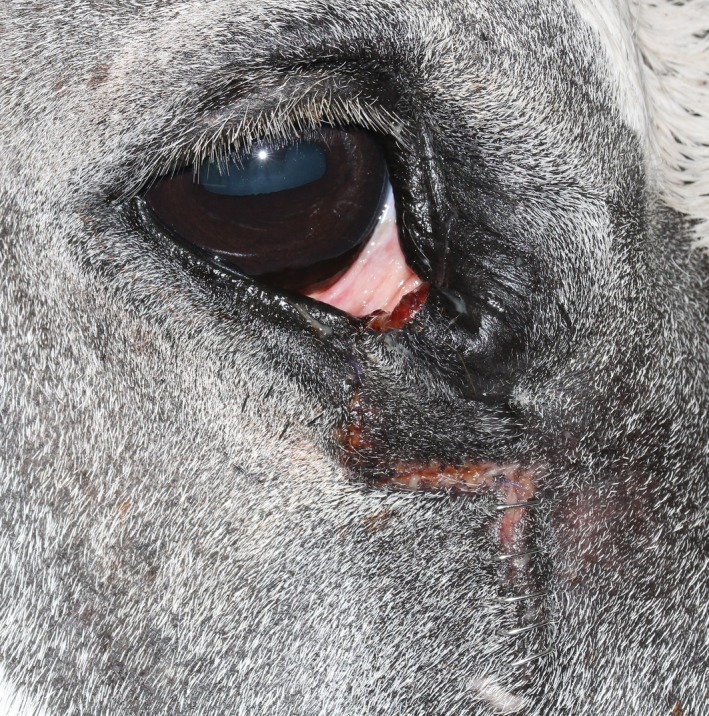
Right eye 10 d postoperatively

**Figure 6:**
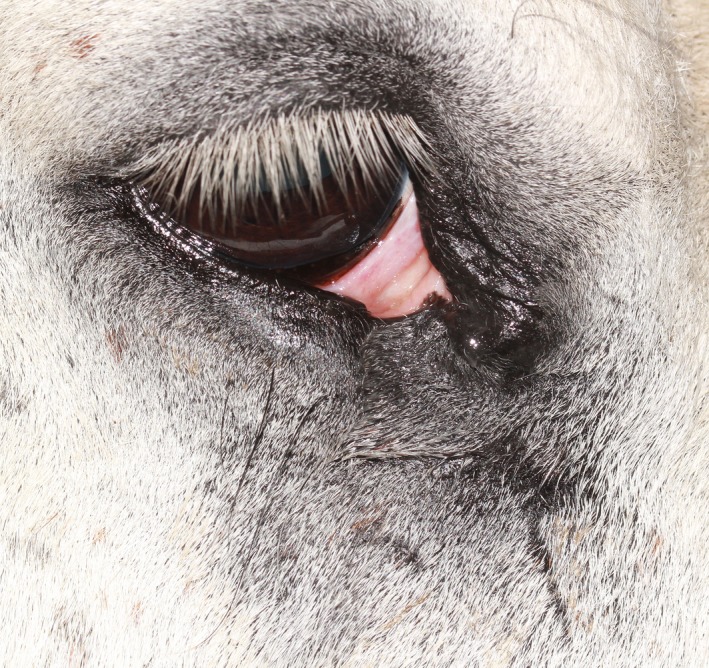
Right eye 1 y postoperatively

Postoperative oral medication consisted of oxytetracycline (Engemycin^®^; MSD Animal Health, Milton Keynes, UK) 8 mg/kg IV q12 h and flunixin (Norbrook, Newry, UK) 1.1 mg/kg IV q12 h intravenously for 6 days. The day after surgery the temporary tarsorrhaphy was removed due to accumulation of discharge. As a slight gap formation occurred on one of the ventral skin incisions away from the eyelids, staples were placed to achieve better apposition of the skin margins. Healing progressed well and the patient was bright and comfortable until discharge 6 days after surgery. The patient was discharged on doxycycline (Karidox^®^; Nimrod Veterinary Products Ltd., Moreton‐in‐Marsh, UK) 10 mg/kg PO q12 h for ten days and suxibuzone (Danilon^®^; Elanco Animal Health, Basingstoke, UK) 2.2 g PO q12 h for 5 days. The patient was stall‐rested for 14 days postoperatively. The protective eye mask was kept in place during this period; the owner was instructed to remove the mask once daily to inspect the wound. The skin staples were removed 14 days after surgery. Re‐examination was performed 10 days, 1 month, and 12 months postoperatively (Figures 5 and 6). A cosmetic repair with normal lower eyelid motility was present. A normal palpebral reflex was present, and the eyelids covered the whole cornea during the blinking action. No evidence of recurrence was observed at the last examination 12 months postsurgery.

## 3 DISCUSSION

The case report describes the novel use of a rhomboid blepharoplasty in a horse following excision of a SCC and cryotherapy of the wound margins. Blanchard and Keller previously described the use of a rhomboid flap for reconstruction of the lateral eyelids in a horse and a cat following removal of eyelid tumors, and of the medial and lateral canthus in two dogs.^17^ The rhomboid blepharoplasty has been described in the human for reconstruction of the medial canthus following surgical excision of basal cell carcinomas.^16^ To the authors’ knowledge, this is the first case where a medial rhomboid blepharoplasty was performed to reconstruct the lower eyelid in a horse.

Direct closure following excision of an eyelid mass using a simple wedge or four‐sided excision is only possible if less than one third of the eyelid margin is to be removed.^18^ Tumor‐free margins of 2 cm have been recommended when attempting to achieve complete excision of squamous cell carcinomas in the horse, but are frequently impossible to achieve if the globe is to be spared.^1^, ^18^ Studies have shown that surgical excision alone carries a high risk of recurrence.^8^, ^9^, ^19^ This is particularly true of eyelid squamous cell carcinomas, although neoplasms affecting the nictitating membrane carry a better prognosis following surgical excision.^8^, ^20^ This is presumably because wider margins can be achieved more frequently.^12^ Several blepharoplasty techniques have been described to facilitate closure of large eyelid defects in the equine. These include the sliding skin graft or "H"‐plasty,^21^ the tarsoconjunctival advancement graft,^14^ the full‐thickness eyelid graft (Cutler‐Beard procedure),^22^ the rhomboid graft,^17^ and the sliding "Z" graft.^14^ The aim of these blepharoplasties is to restore a functional eyelid margin, with minimal complications on the ocular surface and an acceptable cosmetic appearance. In these techniques, a portion of the eyelid margin is replaced with haired skin, which may cause trichiasis. In other species, different blepharoplasty techniques such as the Mustardé technique and lip to lid graft have been described to close large eyelid defects, which aim to maintain a mucocutaneous junction at the eyelid margin.^23^, ^24^ Techniques such as these are limited in the equine, as the periorbital skin is tightly adherent to the underlying fascia and relatively immobile, while the thin eyelids are very delicate. This makes blepharoplasties in horses challenging, and the grafts prone to dehiscence and necrosis.^1^, ^18^ The advantages and disadvantages of each blepharoplasty technique must be considered for every case, in order to choose the one most suitable for the individual patient. The rhomboid blepharoplasty allows closure of a large eyelid defect using haired skin. After a rhomboid graft is performed, the retraction force vectors are expected to be directed alongside the palpebral fissure. This may be an advantage compared to other blepharoplasties, for example, an H‐plasty, where these vectors are expected to develop perpendicularly to the palpebral fissure and potentially predispose to postoperative ectropion and lagophthalmus. Other techniques, such as lateral sliding grafts, rely on the transposition of normal eyelid tissue toward the defect while the haired skin covers an area of corneal surface away from the original defect. A sliding graft of the lateral part of the lower eyelid toward the medial area with a relaxation (burrows) triangle laterally could have been considered in this case. The surgeons prioritized not altering the lateral canthus and the normal lateral part of the lower eyelid. The advantage achieved with the rhomboid technique is that the cornea is shielded by the nictitans membrane from trichiasis, thus minimizing the risk of postoperative corneal irritation. Furthermore, if excision of a tumor is later shown to be incomplete on histopathology, sparing the lateral canthus would allow a sliding lateral blepharoplasty to be performed at a later date. One potential disadvantage of the rhomboid blepharoplasty was that the extensive mobilization of tissue at the medial canthus would require the sacrifice of the nasolacrimal apparatus. Because of the proximity of the tumor to the nasolacrimal puncta, attempting to preserve the nasolacrimal duct was considered likely to increase the risk of recurrence. In this case, loss of the nasolacrimal duct did not cause any clinical signs.

A combination of surgical excision or debulking with adjunctive therapy has been shown on numerous occasions to provide reduced recurrence rates compared to surgery alone.^8^, ^12^, ^25^, ^26^, ^27^ In the literature, various combination therapies have been described alongside surgery for treatment of eyelid SCC. These include photodynamic therapy,^25^ cryotherapy,^8^, ^9^ carbon dioxide laser,^28^ radiation therapy with strontium‐90, iridium‐192, cobalt‐60, radon‐222 or gold‐198,^8^, ^29^, ^30^, ^31^, ^32^ and chemotherapy with mitomycin C or cisplatin.^8^, ^11^, ^33^, ^34^ There is currently insufficient evidence to compare the efficacy of these adjunctive therapies on eyelid SCCs.^12^

For this patient, it was elected to use cryotherapy of the margins following surgical excision of the SCC with 5 mm margins. Cryotherapy can preserve normal structure and function of the tissue after healing.^19^ It is commonly used in combination with surgery for treatment of ocular SCCs, and has also been reported used alone without surgery.^9^, ^35^, ^36^, ^37^ Liquid nitrogen, nitrous oxide or carbon dioxide is used to reduce the temperature of the mass to between −20 and −40°C.^12^ Either a cryoprobe or a cryospray may be used. This treatment is used to target any malignant cells that may remain once the tumor has been excised, although there is potential for significant tissue damage to occur to neighboring tissue.^12^ The advantages of cryotherapy are that it causes reduced tissue scarring and relatively little postoperative pain compared to other therapies.^12^ It is also relatively low in cost.^12^, ^38^

Success rates for cryotherapy of ocular and periocular SCC combined with surgery have been described in the literature. The prevalence of nonrecurrence ranges from 33.3%,^9^ 55%,^36^ to 100%.^37^ Nonrecurrence of periocular SCC was reported in 66.7% of patients with cryotherapy alone.^35^ These studies were all limited by the small number of patients, and it is possible that the success rates were influenced by the position, and thus the behavior, of the SCC. However, it is clear that surgery combined with cryotherapy has potential to be a successful modality for treatment of this disease.

## 4 CONCLUSION

This case demonstrates that a rhomboid blepharoplasty can be used to achieve functional and cosmetic eyelid reconstruction at the medial canthus in the horse.

## CONFLICT OF INTEREST

None declared.

## AUTHOR CONTRIBUTIONS

EJ: prepared the manuscript. SK, CD, BD, and RT: involved in clinical case decisions and edited the manuscript. RT: performed the surgery.
